# Assessment of spatio-temporal evolution trends and driving factors of green development in Harbin-Changchun urban agglomeration

**DOI:** 10.1038/s41598-023-44091-w

**Published:** 2023-10-05

**Authors:** Yang Tang, Yongbo Yuan, Boquan Tian

**Affiliations:** Faculty of Infrastructure Engineering, Dalian University of Technology, Dalian, 116024 China

**Keywords:** Ecology, Environmental social sciences

## Abstract

As China has entered a new stage of high-quality development, clarifying the mechanism and spatial characteristics of green development for urban agglomerations are critical to sustainable development. Based on the data of 11 major cities in the Harbin-Changchun urban agglomeration (HCUA) from 2010 to 2020, this study constructs an evaluation system of green development index (GDI) is composed of four dimensions, i.e. urban green construction (UGC), industrial green development (IGD), resource and environmental carrying capacity (RECC), and technological innovation support (TIS). Furthermore, using the entropy weight method to obtain the weights of evaluation indicators. And then, the comprehensive index calculation is used to evaluate the GDI. The driving factors of each level of GDI are determined by the Pearson correlation coefficient. The results infer some novel findings as follows: (1) the overall tendency of the GDI of the HCUA has gradually increased from 0.358 in 2010 to 0.379 in 2020 which is at the average level. The dimension of TIS shows the highest rate of contribution while IGD and RECC show a fluctuating trend over the time window. (2) The GDI in the HCUA exhibits a patchy clustering differentiation feature that spreads from the central area to the surrounding areas with a “high in the south and low in the north” pattern. Specifically, Changchun, Harbin, and Daqing form an “inverted triangle” structure in geographical location to drive the green development of neighboring areas. (3) The core cities of the HCUA, Changchun, and Harbin, show a much higher level than the other cities. Jilin and Daqing are at the average level, and besides, the rest of the cities of GDI are at the poor level with significant fluctuations in ranking. (4) There are different driving factors between each level of GDI. For cities with good and average levels should focus on protecting resources and the environment. Meanwhile, cities with poor level of GDI need to improve IGD to optimize the urban green structure. Thus, it is suggested to strengthen the flow of factors and implement differentiated strategies to promote coordinated development and spatial clustering.

## Introduction

### Background

With the increasing global environmental resource pressure and energy crisis, the demand for low-carbon and sustainable development has become more urgent. Currently, China has entered a new stage of high-quality development, emphasizing the improvement of energy efficiency and resource utilization, reduction of pollutant emissions, and establishing the direction of resource-saving and environmentally friendly development. According to the 2022 (Environmental Performance Index EPI) report, China’s EPI score ranks 160th out of 180 countries, indicating that there is still a certain gap compared with other countries^1^. As the core engine of sustainable development, green development is based on the model of ecological priority and the common development of the social economy. It is an important way to transform the economy from extensive to intensive, and an effective driving force for China to achieve peak carbon emissions and carbon neutrality.

Urban agglomeration, which accounts for more than 80% of the total population and industries, are key areas where ecological and environmental problems are highly concentrated and have a significant impact on regionally balanced development. They directly affect the progress and implementation of China’s green development^2^. The release of the “National New Urbanization Plan (2021–2035)” further promotes the construction of new urbanization and promotes regional coordinated development. The economic links between cities in urban agglomeration become closer, and the correlation of industrial structure adjustment is higher. In 2018, China further proposed that regional urban agglomeration could play a key role in promoting high-quality development. However, urban agglomeration also generates up to 70% of the total pollution in China, which has a significant negative impact on the economic development and livability of urban clusters.

Regional urban agglomeration green development plays an important role in promoting green development in broader areas and even the whole country. Therefore, correctly understanding the level of green development of urban clusters in China and its influencing factors has important practical significance for improving the regional development coordination mechanism, effectively allocating urban advantages resources, and exploring the difficulties of urban green development.

### Literature review

Green development is an extension of sustainable development. The studies have gone through a process from monolithic to multifaceted. It was first cited in the field of green economy by British scholar David Pierce in 1989, which emphasized the relationship between social development and ecological conditions. At that time, the core of green development was environmental protection^3,4^. In further research, the connotation of green development has been extended from different perspectives, forming a new concept with the core of economic growth, environmental responsibility, and industrial transformation, which has the goal of green development is to maximize social, economic, and ecological coordination and comprehensive benefits^5,6^.

From the perspective of research content, previous studies included the single-factor and the multi-factor of green development. On the one hand, a particular driving factors such as financial economics, industry drivers, and social welfare. Xu et al.^7^ analyzed the different impacts of financial clusters in China’s eastern, central, and western regions on green development which proved that the financial cluster effect in the eastern and central regions is conducive to improving green development. Liu et al.^8^ conducted on the impact of industry drivers and verified the evolution law of regional green development with the different degrees of the cluster in the manufacturing industry. Zhang’s research found that social welfare factors play an important role in influencing the level of green development^9^. Studies about different industries of green development also laid the foundation for future research, such as Bozorgzadeh’s analysis of green development issues from the perspective of water resource allocation^10^. Chang et al.^11^ used the green development framework to explore the path of industrial upgrading. Chen et al.^12^ analyzed the effects of digital transformation of heavily-polluting enterprises under the guidance of green development using total factor productivity as the indicator. On the other hand, a multi-dimensional indicator system based on “economic-social-resource endowment” is used to reflect the regional green development level. Wang emphasized that green development includes the coordinated symbiosis between the economic system, social system, and natural system^13^. Zhang and Sun expanded the indicator system for evaluating green development by including policy systems and quality of life in the social system^14,15^.

Green development evaluation models have been promoted by the rise of sustainability theory and methods. Liu et al.^16^ used the analytic hierarchy process (AHP) to determine the weight of indicators and analyze and compare the green development of different prefecture-level cities. Luo et al.^17^ measured the green development of the Yangtze River Economic Belt with principal component analysis (PCA) to explore the characteristics and differences of green development in the upper, middle, and lower reaches. Wang et al.^18^ selected the TOPSIS model to calculate the green development level of 10 cities in Shandong Province, the results showed that the labor productivity of secondary and tertiary industries is the main obstacle factor for the green development level in this region.

At present, the research objects focus on traditional administrative regions, ranging from an international perspective to countries, provinces, cities, such as OECD countries and Third World countries which have typical economic characteristics^19–22^. China’s national-level urban agglomerations such as the Yangtze River Delta urban agglomeration and the Beijing-Tianjin-Hebei urban agglomeration have also attracted widespread attention from domestic and foreign scholars^23–25^. A small number of regional urban agglomeration studies measure the green development level of cities by calculating specific industries or factors, such as economic factors^26^, agricultural development^27^, and ecological governance^28^, making it difficult to fully reflect the urban agglomeration`s green development condition.

In summary, some limitations of existing studies on green development include the following. To start with, most studies consider economic, social, and ecological factors of indicator systems. Scientific and technological innovation is the fundamental driving force for the green and low-carbon transformation of cities. There has little discussion on factors related to technological innovation development. Next, there are few green developments in China’s regional urban agglomerations. Most focus on countries, provinces, and national-level urban agglomerations, such as the Yangtze River Delta and the Pearl River Delta. Regional urban agglomerations are important economic growth areas, which provide a guarantee for the development of national-level urban agglomerations. However, the lack of regional urban agglomerations` green development is not conducive to the establishment of a unified evaluation mechanism. Finally, studies mainly aim at the index evaluation system and analysis of spatial and temporal characteristics of green development. Hence, the strengths and weaknesses of cities at different levels of green development within urban agglomeration have been neglected.

### Research objectives

**Figure 1:**
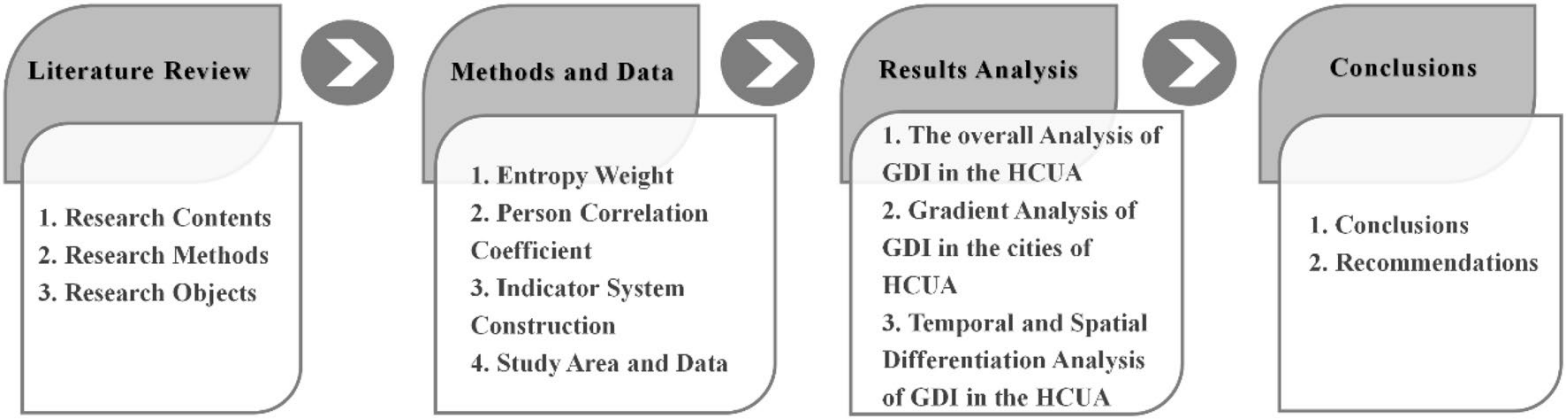
The research process of green development.

To sum up, we selected data, including the year from 2010 to 2020, of 11 cities in the Harbin-Changchun urban agglomeration (HCUA). The green development evaluation system was constructed with 16 indicators in four dimensions: urban green construction (UGC), industrial green development (IGD), resource and environmental carrying capacity (RECC), and technological innovation support (TIS). In the thinking of “whole-local” and “time–space” of the urban agglomeration, we analyze the evolution trend and spatial differentiation characteristics of the green development of HCUA. Combined with the relevant factors, we try to explore the laws and main influencing factors of green development to provide references for the green construction of regional urban agglomerations. Firstly, this study uses the entropy weight method to assign weights to each indicator in the green development evaluation system of the HCUA, reducing the disadvantages of subjective weighting. Secondly, according to the normalized value of each indicator and the corresponding weight, the multi-objective linear sum method is used to calculate the evaluation value of the green development level of HCUA. Next, SPSS statistical software is used to obtain the Pearson correlation coefficient of GDI and each dimension, further exploring the correlation between green development and each related factor. Finally, use Geoda software to visually characterize the differences in GDI between cities. The research process is shown in Fig. 1.

## Methods and data

### Connotation of urban green development

At present, there is a lack of clear definitions and standards on the connotation of green and low-carbon urban development, which can be summarized in three aspects. First of all, some studies advocate a paradigm shift in urban development from a holistic sustainability perspective, emphasizing four aspects of natural resources, land use, and transportation, energy, pollution, and waste^28,29^ What’s more, people-centered concepts are emphasized, including housing, employment, and transportation activities, which improve urban functions and enhance livability and sustainability^30^. At last, the performance of new urbanization is the synergistic development of material, spiritual, political, and ecological, which promote the harmonious development of human beings and nature^31^. Therefore, urban green development includes measures to mitigate pollution and emissions such as rational planning of urban layout, optimization of urban industrial structure, development of efficient transportation system, promotion of green and low-carbon buildings, optimization of energy structure, and adaptation to climate change with improving infrastructure and enhancing governance capacity^32–34^.

The theory of environmental economics provides an important reference for how to correctly understand and deal with the relationship between human, nature and society in the process of green development, and also provides an important idea for the improvement of the current concept of green development. Many views and conclusions of environmental economics theory are related to green development, the most important is the environmental Kuznets hypothesis. According to the Kuznets hypothesis, with the continuous improvement of per capita income, the continuous progress of scientific and technological level, and the continuous optimization of industrial structure will be conducive to the improvement and enhancement of environmental quality. However, this conclusion may lead people to fall into the misconception which is environmental problems will be solved with the improvement of economic development level automatically. At the same time, the environmental Kuznitz curve illustrates the importance of economic development. Therefore, the green development of the city can be summarized in four aspects: low pollution, low energy consumption, high efficiency and high output which known as “two low and two high”. Therefore, when discussing the level of urban green development, we should analyze the level of green development in different regions from the angle of ecological and economic balance.

### Assessment method of green development

#### Multi factor comprehensive evaluation method

The evaluation of green development index (GDI) with three steps, which including data standardization, weight determination and GDI estimation.

1. Data standardization

Dimensionless standardization was employed to eliminate the influence of different dimensions of the original index data. For the treatment of income indicators (the greater the better), see Eq. (1); for the treatment of cost indicators (the smaller the better), see Eq. (2):

1 $$x_{ij}^{ * } = \frac{{x_{ij} - \min (x_{j} )}}{{\max (x_{j} ) - \min (x_{j} )}},$$

2 $$x_{ij}^{ * } = \frac{{\max (x_{j} ) - x_{ij} }}{{\max (x_{j} ) - \min (x_{j} )}},$$

where x^*^_ij_ is the normalized of indicators. The subscript *i* denotes the year, and *j* represents indicator; Max (*x*_*i*_) and min (*x*_*j*_) refer to the maximum and minimum values of the indicator *j*, respectively. Then, different attributes of the basic index are consistent and comparable.

(2) Weight determination

To improve the objectivity of the green development evaluation, considering the complexity of the urban system and the uncertainty of the indicator, the weight was determined by the entropy weight method. Firstly, the entropy value *e*_*j*_ of the indicator *j* is calculated as Eq. (3):

3 $$e_{j} = - \frac{1}{\ln n}\sum\limits_{i = 1}^{n} {p_{ij} \ln p_{ij} } ,$$

where *p*_*ij*_ represents the proportion of the indicator *j* in the year of* i*.

Next, the difference coefficient* g*_*j*_ of indicator *j* is calculated according to Eq. (4). The larger the entropy value *e*_*j*_, the smaller the difference value, and the less important the indicator.

4 $$g_{j} = 1 - e_{j} ,$$

The final step is to calculate the entropy weight *w*_*j*_ for the indicator* j* as Eq. (5):

5 $$w_{j} = \frac{{g_{j} }}{{\sum\limits_{j = 1}^{n} {g_{j} } }}.$$

(3) GDI estimation

The normalized values and weights obtained from the above steps are linearly summed to calculate the green development index for each dimension in the evaluation system using Eq. (6), and then the comprehensive green development index for each city in the HCUA is calculated using Eq. (7).

6 $$z_{is} = \sum\limits_{j}^{n} {x_{ij}^{ * } } w_{j} ,$$

7 $$GDI_{i} = \sum\limits_{s = 1}^{4} {{\text{z}}_{it} ,} \,\,\,\,(i = 1,2,3, \ldots ,I;j = 1,2,3, \ldots ,J)$$

where *z*_*is*_ represents the green development of each dimension in the year *i*; *n* is the number of indicators. The grading of the GDI is the result based on a number of objective indicators and data. At present, the green development ratings of the cities of China have not achieved uniformity. Equidistant Quintiles method is applicable to the comprehensive evaluation of multiple indicators, which provides a better understanding and comparison of the trends and characteristics of green development. The GDI ranges from 0 to 1, and the standard is divided into five levels^36^, as shown in Table 1.

**Table 1:** Standards of green development index.

	[0, 0.2)	[0.2, 0.4)	[0.4, 0.6)	[0.6, 0.8)	[0.8, 1)
Level of GDI	V	IV	III	II	I
Description	Poor	Inferior	Average	Good	Excellent

#### Pearson correlation coefficient evaluation method

Generally, the description of multivariate relationships can be expressed with functional or correlation relationships. The former is the existence of a definite correspondence between two variables, and the latter refers to a changing pattern within a certain range without a clear corresponding relationship. In this study, correlation analysis of variables can measure the degree of association between different dimensions and green development, where the Person correlation coefficient is used to visualize the correlation between variables^36^.

The Pearson correlation coefficient for n pairs of data (*a*_*i*_, *b*_*i*_) (*i* = 1,2,…,n) calculated Eq. (8).

8 $$r = \frac{{\sum\limits_{i = 1}^{n} {(a_{i} - \overline{a})(b_{i} - \overline{b})} }}{{\sqrt {\sum\limits_{i = 1}^{n} {(a_{i} - \overline{a})^{2} \sum\limits_{i = 1}^{n} {(b_{i} - \overline{b})^{2} } } }^{{}} }}.$$

Among them, $\overline{a} = \sum\nolimits_{i = 1}^{n} {a_{i} }$, ${\overline{\text{b}}} = \sum\nolimits_{i = 1}^{n} {b_{i} }$, and the range of the Pearson correlation coefficient is |r|≤ 1. The positive value of r indicates two variables change in the same direction when the independent variable increases. When one variable increases or decreases, the other variable also increases or decreases accordingly. The negative value of r indicates a negative relationship between two variables. Table 2 shows the range of values of the Pearson correlation coefficient and the criteria for the correlation of variables.

**Table 2:** Standards for Pearson correlation coefficient.

Pearson correlation coefficient value	Correlation relationship	Value range	Degree of relationship
|r|< 1	A positive value of r indicates a positive correlation, while a negative value of r indicates a negative correlation	0.95 <|r|< 1	Significant correlation
		0.8 <|r|< 0.95	Highly correlated relationship
		0.5 <|r|< 0.8	Moderate correlation
		0.3 <|r|< 0.5	Low correlation
		0 <|r|< 0.3	Weak correlation
|r|= 1	Perfect correlation		
|r|= 0	Uncorrelated		

The sample correlation coefficient is almost optimal for correlations between variables that are asymptotically unbiased and valid. Pearson correlation coefficient requires that the data obey a normal distribution in which the data are equally spaced, at least in the logistic range. Moreover, the effect of outliers is removed^38^. If two random variables are independent of each other, they must not be correlated. Conversely, uncorrelated variables are not necessarily independent of each other. When the linear correlation is negated, there may be other correlations between the variables that are not necessarily uncorrelated. If (a, b) obeys normal distribution, then uncorrelated is equivalent to mutually independent.

### Construction of indicator system of GDI

Urban green development takes into account the optimization of construction mode, rational allocation of resources, and motivation mechanism to achieve a balanced development of the social-life-consumption pattern^39^. Most of the internationally recognized evaluation indicator systems are aimed at the national scale, which lacks compatibility with the actual condition in China. Currently, the indicator systems suitable for evaluating green development at the urban scale in China include the “Green Development Indicator System”, “Ecological Civilization Construction Assessment Target System”, and “Green City Evaluation Indicators (consultation version)”, which cover economic, municipal, residential life, resource, and ecological aspects. Based on the principles of systematic, operable, and accessible index construction, we constructed an indicator system that consider two aspects. On the one hand, referring to the index system introduced above. On the other hand, combining the particularity of the HCUA and the results of references^39–42^, the indicator system of GDI includes four dimensions: urban green construction (UGC), industrial green development (IGD), resource and environmental carrying capacity (RECC), and technological innovation support (TIS).

**Figure 2:**
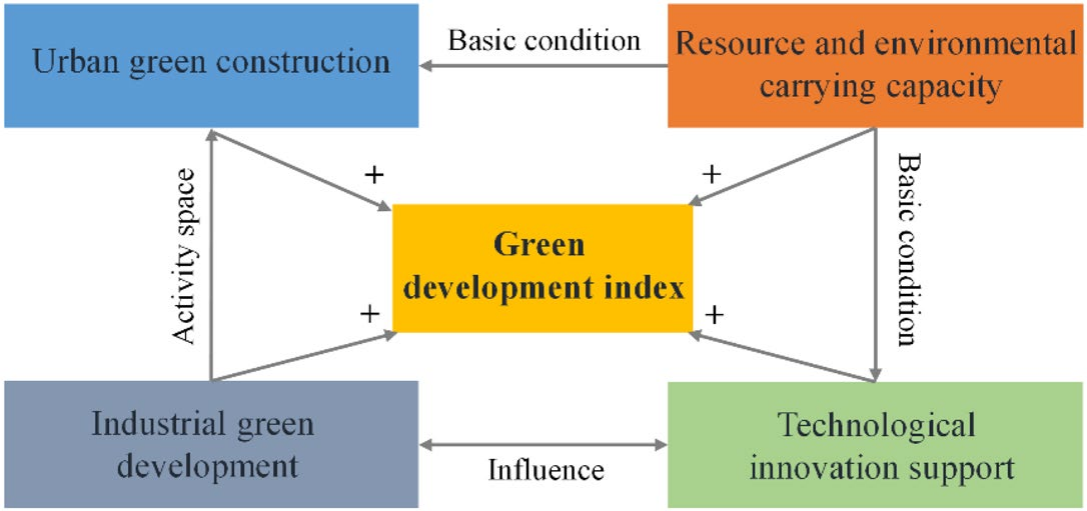
Influence relationships among dimensions of green development.

Figure 2 shows the interconnection between four evaluation dimensions of green development. Firstly, urban green construction drives the industrial green development. The construction within cities, by integrating urban transportation, green architectural environments, and resource utilization, can significantly reduce energy consumption and environmental pollution, thereby enhancing urban livability and sustainability. It provides superior urban environment and production conditions for the industrial green development. Secondly, industrial green development contributes to the enhancement of urban green construction, to adopt environmentally friendly production processes to reduce pollution emissions and improve the efficiency of resource utilisation. Moreover, resource and environmental carrying capacity is the basis for green development. Both green urban development and innovative activities in science and technology require adequate resource supply and environmental capacity to support them. Insufficient resource and environmental carrying capacity can result in irreversible environmental damage. Finally, technological innovation support is an important driving force for green development. Science and technology innovation can provide advanced technological means for urban green construction and industrial green development, which lead to a more efficient development path and provide innovative solutions in all dimensions.

Specific evaluation indicators of GDI are shown in Table 3. To avoid data distortion caused by the population and geographical area of different regions, we used the per capita, utilization rate, and percentage to construct the evaluation indicator system.

1. Urban green construction. It reflects the continuous improvement of urban municipal and transport systems to reduce environmental hazards. Per capita public transportation ownership was chosen to indicate the building of green transport, which helps to improve urban transport efficiency and air quality^44^. Considering the change in construction mode, a high proportion of green buildings can accelerate the process of low-carbon development, reduce urban water consumption and improve the disposal of domestic waste^45^. Per capita water use and the rate of non-destructive disposal of domestic waste represent the impact and response of building activities, respectively^44,46^.
2. Industrial green development. Increased social and economic scale not only improves the income and living standards of urban residents but also causes resource consumption and waste emissions. Consider the sustainability and environmental friendliness of urban industries from a number of perspectives, including economics, resource utilisation and environmental protection. Per capita gross domestic product (GDP) reflects the economic scale, with higher GDP indicating stronger industrial development^46,47^. Land area used for construction per unit of GDP measures the consumption of land resources, that is, the efficiency of economic output and the dependence on natural resources^46,47^. Electricity consumption per unit of GDP indicates the consumption of energy in industrial production, and a lower value means more energy-efficient industrial production^48^. The higher comprehensive utilization rate of industrial solid waste, the better the recycling of resources and the lower the load on the environment^46,48^.
3. Resource and environmental carrying capacity. The ecological environment is the foundation for supporting green development. The capacity green coverage rate of built-up areas is an indicator that measures the proportion of green space^46,47^. High green space coverage helps to enhance the urban ecological environment, absorb carbon dioxide, improve air quality and reduce the heat island effect. Per capita urban green space reflects the quality of the urban living environment, which provides more space for green recreation and outdoor activities, contributing to improved life satisfaction and health of residents^46^. Energy consumption per unit of GDP and sulfur dioxide emissions per unit of GDP indicate the use and consumption of energy for economic benefits, respectively^46,49^ The lower values are, the higher the resources and environmental carrying capacity, which is consistent with the goal of green development.
4. Technological innovation support. It reflects the innovative strength and development potential. Per capita technological expenditure and number of students enrolled in ordinary colleges and universities affect research and development inputs^47,50^. Per capita number of patents granted indicates the transformation of innovations and the protection of intellectual property rights^50^. Number of major science and technology infrastructure construction provides good hardware conditions for scientific research^47,50^. These indicators can reveal the strength and development direction of the technology innovation.

**Table 3:** Evaluation indicator system for GDI of the HCUA.

Target layer	Criterion layer	Indicator layer	Unit	Weights
Green development index (GDI)	Urban green construction (UGC)	Per capita public transportation ownership ( +)	80 person per bus	0.048
		Per capita water consumption (–)	t	0.029
		The proportion of green buildings ( +)	%	0.032
		Harmless treatment rate of domestic garbage ( +)	%	0.026
	Industrial green development (IGD)	Per capita gross domestic product (GDP) ( +)	Yuan	0.060
		Land area used for construction per unit of GDP (–)	Km^2^	0.014
		Electricity consumption per unit of GDP (–)	kW·h	0.042
		The comprehensive utilization rate of industrial solid waste ( +)	%	0.023
	Resource and environmental carrying capacity (RECC)	The capacity green coverage rate of built-up areas ( +)	%	0.036
		Per capita urban green space ( +)	m^2^	0.063
		Energy consumption per unit of GDP (–)	Tons of standard coal	0.059
		Sulfur dioxide emissions per unit of GDP (–)	t/100 million yuan	0.025
	Technological innovation support (TIS)	Per capita technological expenditure ( +)	100 million yuan	0.173
		Number of students enrolled in ordinary colleges and universities ( +)	Ten thousand person	0.097
		Per capita number of patents granted ( +)	pcs	0.142
		Number of major science and technology infrastructure construction ( +)	pcs	0.132

“ + ” indicates benefit indicators, and “–” indicates cost indicators in the table.

### Study area and data sources

**Figure 3:**
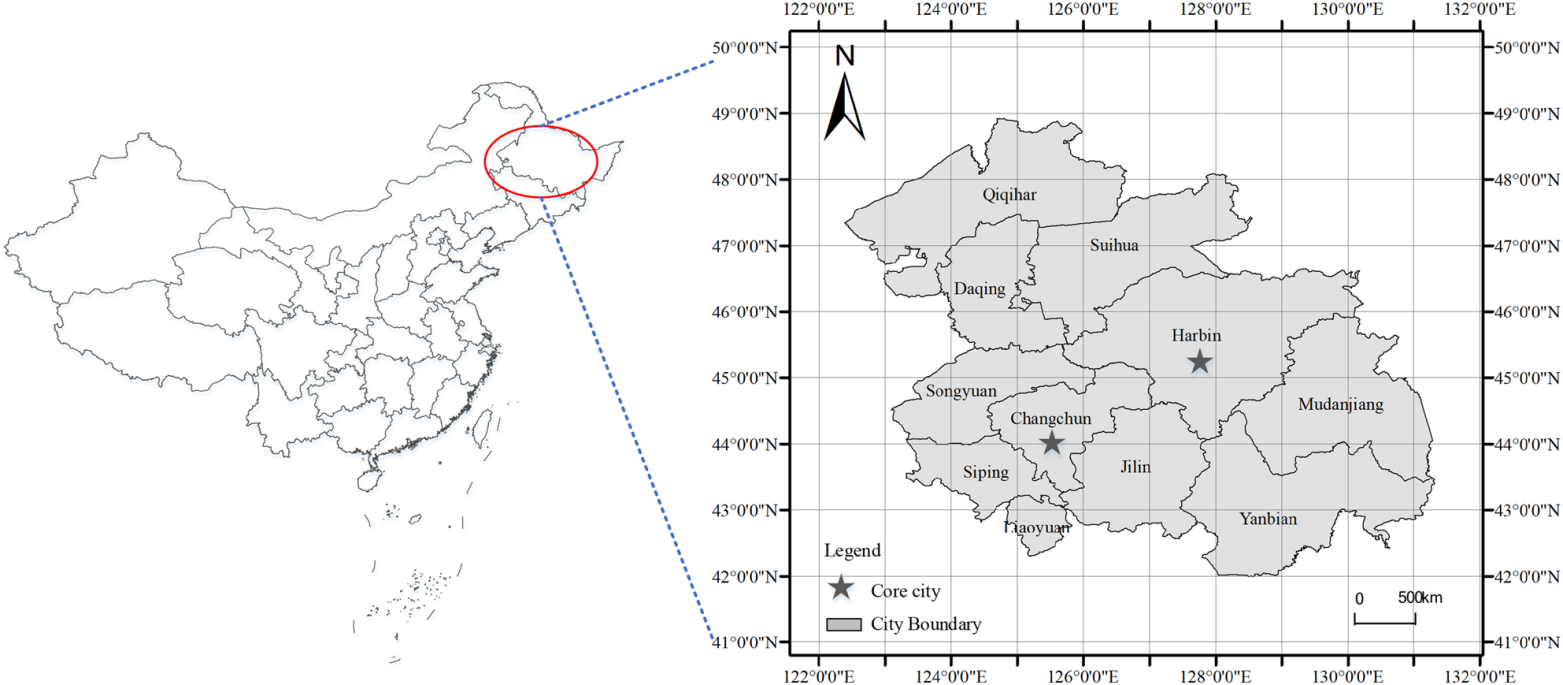
Location and range of the HCUA.

The HCUA, as a priority development area of nine regional urban agglomerations, was proposed in “China’s Thirteenth Five-Year Plan”. The HCUA is the gateway to China’s northeast Asian export-oriented economic construction, which includes Harbin, Daqing, Qiqihar, Suihua, and Mudanjiang of Heilongjiang Province as well as Changchun, Jilin, Siping, Liaoyuan, Songyuan, and Yanbian of Jilin Province. In terms of geographical structure, the HCUA is a typical dual-core urban agglomeration, covering 26,400 square kilometers of national territorial area. Then, the “Development Plan of Harbin-Changchun urban agglomeration” was officially approved by the State Council in the year of 2016, which emphasized the strategy of helping the HCUA into a livable and green urban agglomeration with significant influence and competitiveness. At present, the HCUA has entered a transformation stage driven by multiple cycles of industrial development, modern services, and ecotourism. Exploring the regional green development index and spatial characteristics will balance the economic development of the northeast area. The location and range of HCUA are shown in Fig. 3.

According to the indicator system, the data were collected from statistical yearbooks, statistical bulletins, environmental status bulletins, and the state intellectual property office of China. Data sources are shown in Table 4.

**Table 4:** Data sources.

Data types	Data sources
Socioeconomic data	Statistical Yearbook of Jilin and Heilongjiang Provinces (2011–2021), City Construction Statistical Yearbook of Jilin and Heilongjiang Provinces (2011–2021), Statistical Bulletin of National Economic and Social Development (2011–2021)
Population data	City Statistical Yearbook
Technological innovation data	State Intellectual Property Office of China (https://www.cnipa.gov.cn/)
Resource and environmental data	Environmental Statistical Yearbook (2011–2021), Environmental Status Bulletin (2011–2021)

## Results analysis

### Temporal evolution of green development

#### Overall analysis of the GDI in the HCUA

**Figure 4:**
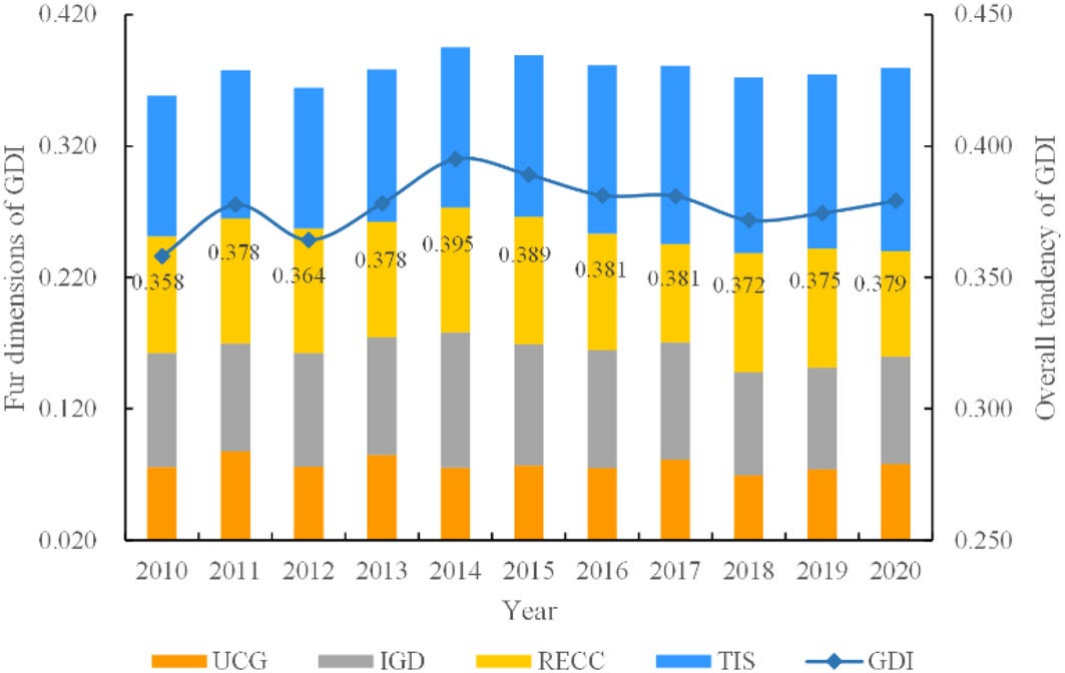
The overall tendency of the GDI in the HUCA during 2010–2020.

Figure 4 indicates the overall tendency of the green development index and the contribution rate of each dimension of the whole HCUA from 2010 to 2020. The overall green development index is obtained by adding the values of each city of HCUA and taking the average value. The analysis shows that the whole HCUA is at the average standards, which has shown an overall upward trend during the studied time window. The GDI increased from a value of 0.358 in 2010 to 0.379 in 2020, with a growth rate of 5.87%. In terms of stages, the GDI showed a trend of rising and then falling in 2010–2012, which was due to the increased investment in infrastructure construction of industrial development. While energy conservation and emission reduction were neglected, partly. During the period of 2012–2014, GDI increased rapidly from 0.364 to 0.395, with an increase of about 8.52% for two reasons. On the one hand, local government is constantly optimizing the industrial structure to promote regional science and technology construction. On the other hand, the promotion of energy conservation and environmental protection policies has stabilized the RECC of HCUA. The urban construction activities in HCUA increased from the year 2014 to 2018, which led to sudden increases in the pressure on resources and environment. As a result, GDI continued to decline to a value of 0.372. With the determination of the HCUA’s key regional strategic positioning, the GDI tended to be in a benign and stable state from 2018 to 2020.

From the point of view of the four dimensions that affect green development, TIS has the highest contribution to green development, with an increased value of 29.77% over the decade, which showed a trend of gradual increase year by year. Owing to the strategy of “double innovation”, the innovation infrastructure in HCUA has been initially developed, increasing from a value of 0.107 in 2010 to 0.123 in 2015, which indicates that the conversion of scientific and technological innovation achievements and industrial restructuring is gradually enhanced. The determination of the 18th national congress of the communist party of China on the core position of science and technology innovation has led to the rapid growth stage of TIS, with the value reaching 1.39 in 2020 and increasing the contribution rate by 6.751%. According to the indicator data, the per capita number of patents granted increased rapidly from a value of 1.085 in 2010 to 4.341 in 2020, indicating that more and more scientific and technological progress enhances the innovation ability of HCUA. The IGD has shown complex fluctuations by the year 2015, and the high-quality economic development stage brings a steady growth of GDP. As the development pattern of resource-based in HCUA has not been completely transformed, economic development still relies on some high-energy-consuming industries as a result of high industrial solid waste emissions and energy consumption. The elements of industrial green development show a trend of rising first and then falling. The trend of RECC is similar to IGD on account of the consumption of resources and environment with economic construction activities, which led to a certain extent restricts the green development of urban agglomerations. Local government has increased investment in urban municipal and transportation infrastructure construction to promote effective green construction in HCUA, with UGC elements reaching 0.078 in 2020, an increase of about 2.63%.

#### Analysis of the evolution trend of GDI

**Figure 5:**
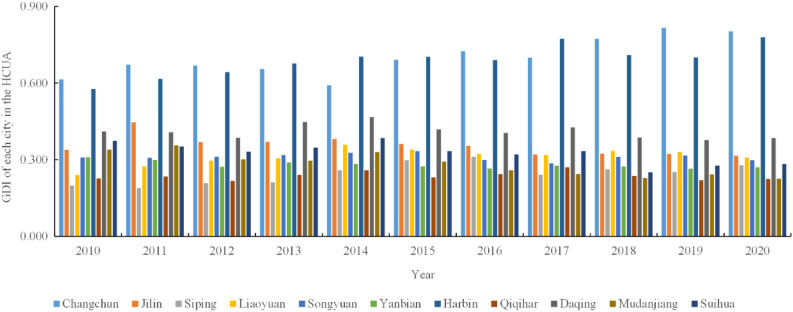
The GDI of the cities in HCUA during 2010–2020.

Figure 5 shows significant differences in the GDI among the 11 cities in HCUA. From the trend perspective, Changchun, Jilin, Siping, Liaoyuan, Songyuan, Harbin, and Qiqihar showed a fluctuating upward trend, while Yanbian, Daqing, Mudanjiang, and Suihua showed a downward trend. During the period from 2010 to 2020, the maximum and minimum values of the GDI have significant differences, which are Changchun (0.816) in 2019 and Siping (0.189) in 2011, respectively. The GDI of each city includes three levels, namely good, average, and inferior, without excellent and poor levels. Changchun and Harbin, as the core cities of HCUA, GDI are far ahead of other cities in the top two positions. They belong to a good level of the green development index, which values are 0.803 and 0.779 in the year 2020, respectively. The growth of core cities is 30.78% and 35.01%, indicating the central region concentrates on the advantages of production factor acquisition, resource allocation, and infrastructure construction. Technological achievements have gradually achieved green transformation, which reflects the regional dominant role of Changchun and Harbin. In addition, Daqing and Jilin are the sub-growth pole cities in the HCUA with the average level. Specifically, Daqing is more successful in its transition from oil and gas resources as a pillar industry to high-quality development. The development direction of digitalization achieves industrial structure optimization, which reduces the consumption of the urban environment and the impact of atmospheric pollution. The rest of the cities of Liaoyuan, Songyuan, Siping, Suihua, Mudanjiang, Qiqihar, and Yanbian are at the inferior level of GDI.

#### Analysis of the changes in ranking of GDI

**Figure 6:**
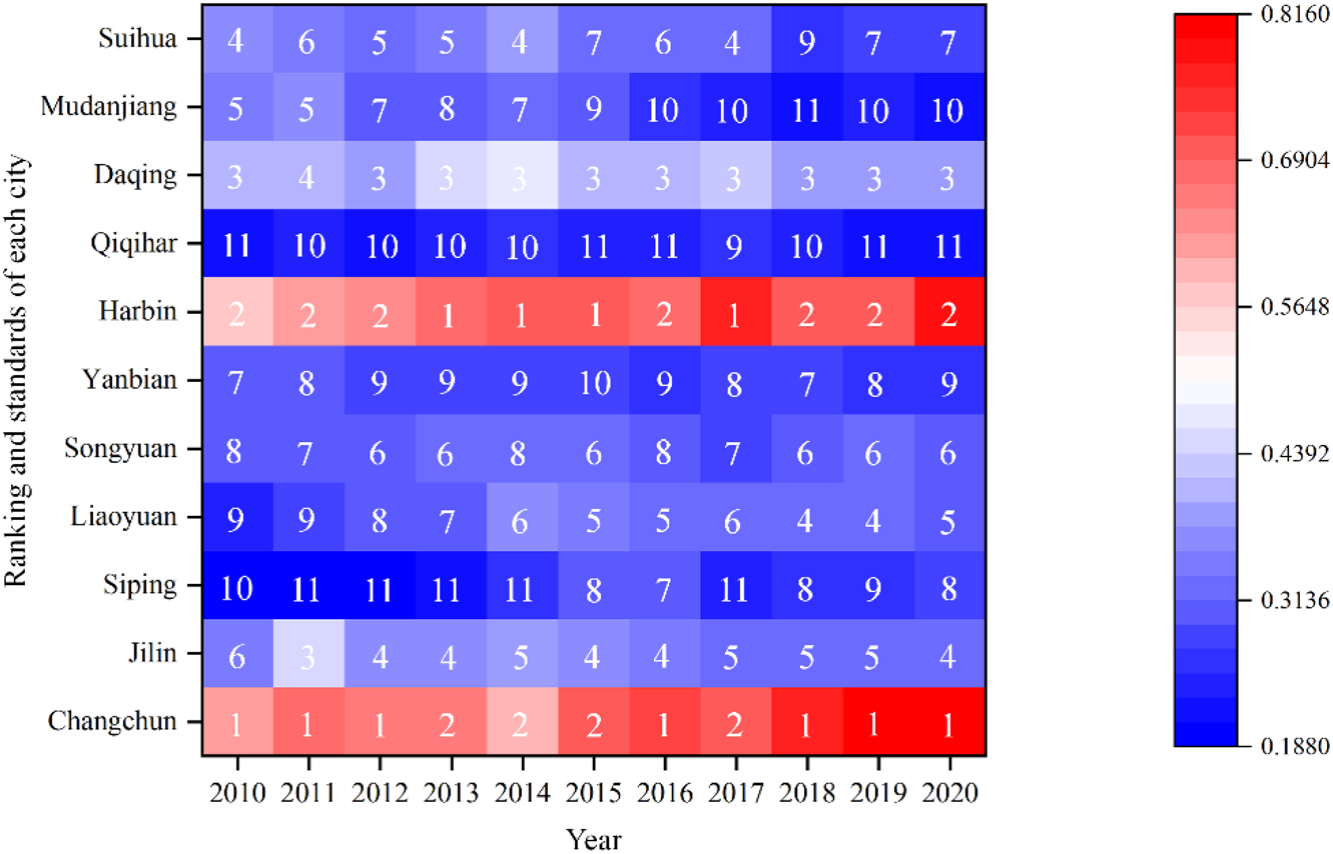
The changes in ranking and standards of GDI of each city in HCUA during 2010–2020.

According to the green development level and ranking of each city in the HCUA from 2010 to 2020, as shown in Fig. 6. It can be seen that Changchun and Harbin entered the excellent level of GDI in 2019 and 2020, respectively. Little change in ranking for Daqing and Jilin, with the former in third place steadily and the latter in sixth place in 2010, then alternating between fourth and fifth place. The rankings of other cities have fluctuated greatly and all are in the lower levels. Among them, the resource-based cities of Liaoyuan and Songyuan show similar changes, with their rankings improving by four and two places respectively. The GDI of Mudanjiang, where tourism is the main industry, slips from fifth place in 2010 to tenth place in 2020. Due to the lack of moderate and innovative development of natural landscapes, resource consumption increases year by year, resulting in a continuous decrease in the carrying capacity of resources and the environment. In addition, Siping improved from the bottom to eighth place, which has strong potential for green development.

**Figure 7:**
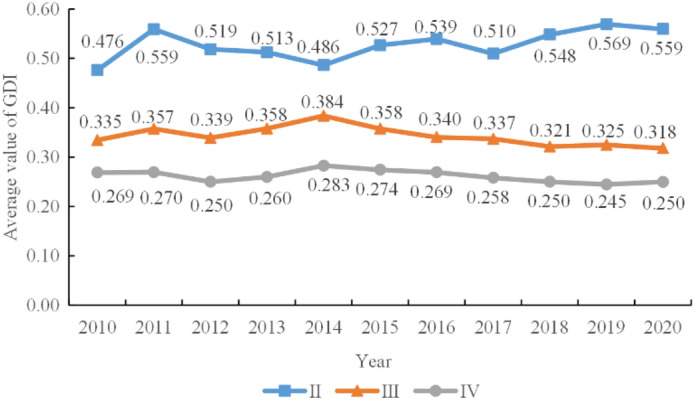
The average value of GDI for each standard in HCUA during 2010–2020.

To explore the characteristics of the various levels of GDI, the average of each level of city in HCUA during 2010–2020 is shown in Fig. 7. First of all, the average value of GDI in level II (good) cities falls between 0.5 and 0.6, which is significantly higher than in other cities and increases year by year. Next, the cities in level III (average) of GDI are between 0.3 and 0.4, with the value increasing slightly to 0.384 in 2012, decreasing in the following years. Last but not least, level IV (inferior) account for about half of the total number of cities in HCUA with dissatisfied GDI, which has poor performance in urban green construction and innovation compared to other cities. The trend of level IV change is similar to that of level III cities.

The absolute and relative gaps in green development levels between different levels of cities are gradually widening. On the one hand, the GDI of level II (0.476) cities was 0.142 higher than that of level III (0.335) cities and 0.208 higher than that of level IV (0.269) in 2010. On the other hand, the difference in GDI between level II (0.559) and level III (0.318) was 0.241, and the difference between level II and level IV (0.250) cities was 0.309 in 2020. As each level of urban development has its focus, the differences in various aspects such as economic development and social factors have formed the divergent characteristics of urban green development.

### Spatial differentiation characteristics of green development

**Figure 8:**
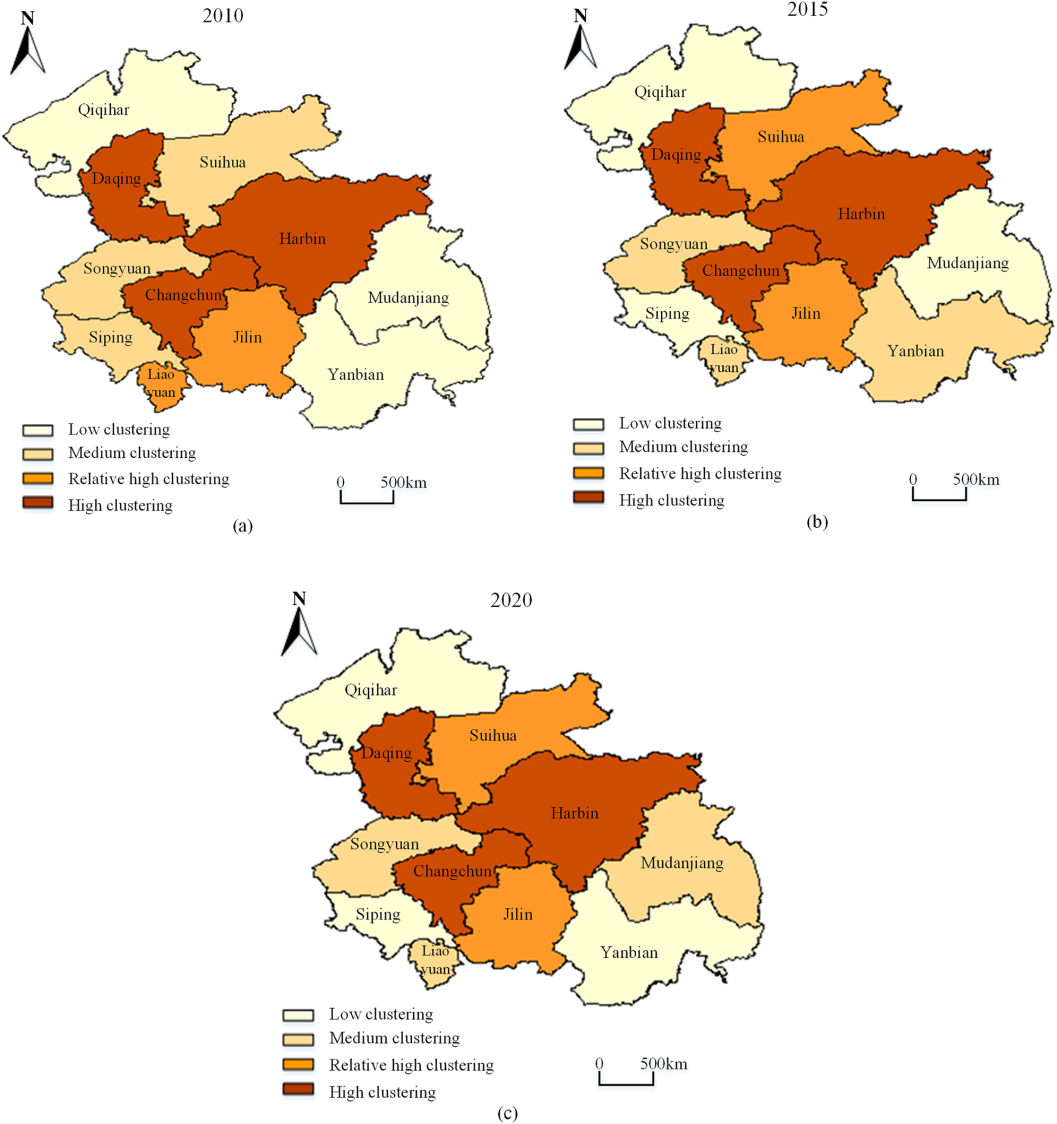
The temporal evolution and spatial differentiation of the GDI in the HCUA. (**a**–**c**) are spatial and temporal differentiation maps of GDI in 2010, 2015 and 2020.

To further explore the spatial distribution characteristics of the GDI in the HCUA, the cluster map function of Geoda1.2.0 software was used to characterize the geographic location differences through adjacency geographic weights. Figure 8 shows the clustered and discrete distributions of GDI by the years 2010, 2015, and 2020, respectively, in which the spatial clustering situation is divided into four different levels. The overall changes of GDI are characterized by the spatial differentiation of fragmented agglomerations that “gradually decrease from the center to the periphery”, that is radiating from Changchun and Harbin to sub-center cities in surrounding areas. This “south high, north low” feature characteristic similar is to the comprehensive benefits of the northeast in China.

From the perspective of the regional area, the most significant clustering of GDI is concentrated in the center of the HCUA in 2010, where the effect is more pronounced in the northern and eastern regions. Changchun, Harbin, and Daqing formed an “inverted triangle” structure in their geographical location with high clustering characteristics. Cities around the core, such as Jilin, Songyuan, and Suihua are driven by moderate clustering characteristics. Whereas, Qiqihar, Mudanjiang, and Yanbian, which are geographically located at the edge of the HCUA, do not have obvious clustering of the GDI. Compared with 2010, the clustering degree of Suihua was raised by one level in 2015, and Yanbian also showed a certain spatial clustering with the impetus of Jilin.

**Figure 9:**
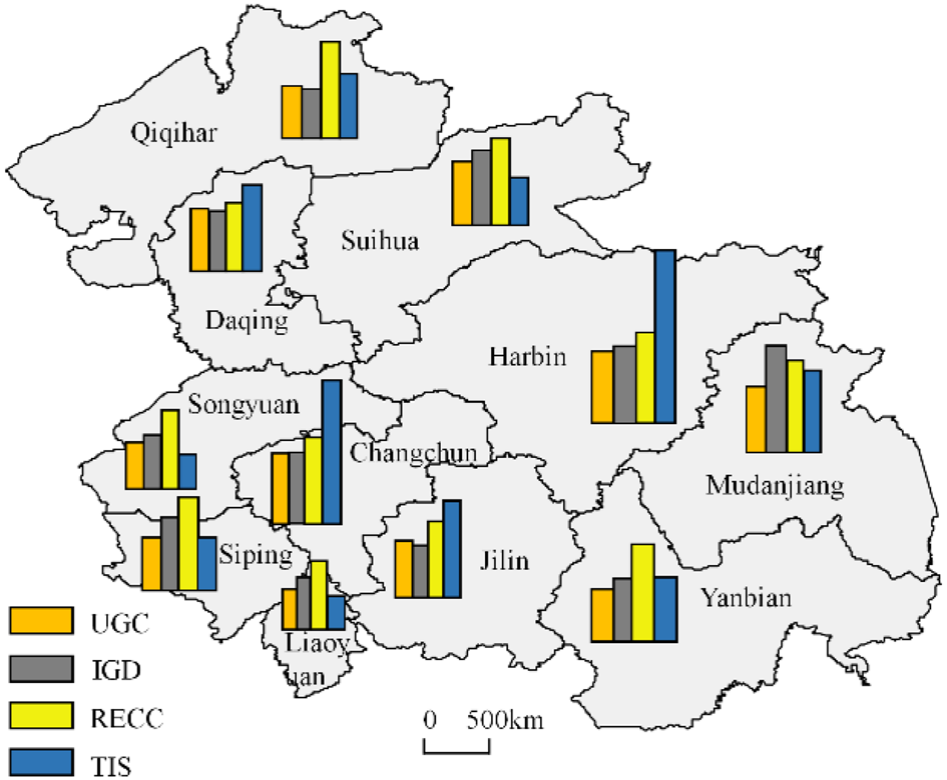
Spatial distribution of four dimensions of GDI in HCUA.

From the perspective of the spatial concentration of resources, the central part of HCUA has concentrated resources such as science, technology, and education, so the trend of polarization is obvious (Fig. 9). The number of major scientific infrastructures and the education level is significantly higher than in other areas, which are important influencing factors of the GDI of Changchun and Harbin. Meanwhile, major scientific infrastructure is spatially directed, and there is a tendency for science and technology resources to spread to the cities of Jilin, Mudanjiang, and Daqing, indicating that the resource factor growth model of the HCUA is transformed to rely on technology. There is little spatial pattern difference in RECC, with southern cities higher than northern cities, that is, Jilin province is better than Heilongjiang province. One reason is the natural endowment advantages of HCUA. Another is the HCUA is dominated by economic development and is still strongly affected by resource and environmental pressure and labor cost. Therefore, it shows the opposite trend of industrial development and resource consumption.

### Analysis of driving factors of green development for each level

The level of green development is determined by various dimensions of factors. Table 5 shows the Pearson correlation coefficients between the GDI and four dimensions of the HCUA, which performs different correlations in each level. Among them, the results of level II cities indicate there is a strong correlation between GDI and two dimensions named UGD and TIS with the value of 0.709** and 0.697**, respectively. The correlation coefficients of the rest of the two dimensions are much lower than UGD, which are IGD (0.188) and RECC (0.120). The performance of level III cities is similar to that of level II cities, while the Pearson correlation coefficient for the TIS dimension is lower than that for level II cities by 0.016. That is, the development patterns of level II and III cities are dominated by TIS. There is little difference in Pearson correlation coefficients of each dimension in the first two levels, which refers the integration of social resources and regional allocation in the HCUA is gradually improving. Specifically, the Pearson correlation coefficient between GDI and RECC for level IV cities is 0.720**, indicating that green development at this level still mainly depends on the regional resource-environment endowment. Hence, more attention should be paid to the utilization of resources and environmental protection.

**Table 5:** Pearson correlation coefficient between each standard of GDI and four dimensions.

	UGC	IGD	RECC	TIS
II	0.709**	0.188	0.120	0.697**
III	0.553**	0.406	0.130	0.671**
IV	0.683**	0.230	0.720**	0.456**

**Denotes a significant correlation at the 0.01 level.

## Discussion

This paper proposes a quantitative evaluation system for the level of green development, aiming to guide the coordinated development of urban economy and resource environment. It provides feasible measurement indicators for the determination of sustainable urban agglomeration development model. The measurement of green development is mainly divided into single drivers and comprehensive evaluation. In the single driver, the value of ecological services is usually used as the evaluation standard^50^. In the multidimensional evaluation system, the focus is on the development of the coupled interaction of “society-economy-resource-environment”^51^ On the basis of previous studies, this paper considers to add relevant indicators of technological innovation support to the evaluation system.

From the perspective of each city, the level of green development is highly variable. The core cities Changchun and Harbin have a higher level of green development, which indicates that the core resources of the city cluster are concentrated in these areas. This phenomenon is consistent with Dai’s findings that the concentration and rational allocation of core resources give city clusters greater development potential, which is in line with the basic characteristics of city cluster development^52^. Therefore, the development of city clusters should focus first on the promotion of core areas, and then gradually drive the development of neighbouring cities.

From the perspective of spatial distribution, the Harbin-Changchun urban agglomeration is at a medium level of green development with improvement in the overall degree, which the radiation-driven effect still needs to be further enhanced. While fewer studies currently focus on the drivers for upgrading the sub-growth pole cities. This paper finds that the sub-growth cities of Jilin and Daqing have prioritised the development of scientific and technological innovation capabilities and the stabilisation of resource and environmental carrying capacity. This helps to form a complementary and mutually reinforcing relationship with the core cities, and enhances the mobility of regional resources in the Harbin-Changchun urban agglomeration. 

## Conclusions and recommendations

### Conclusions

This study constructs an evaluation index system for green development from four dimensions UGC, IGD, RECC, and TIS. Based on the data of indicators from 2010 to 2020, the green development level and spatial differentiation of the HCUA are evaluated and analyzed. The main conclusions are as follows:

1. In the time evolution of the GDI, the HCUA shows an overall trend of first rising, then falling, and then rising again, and is at an average level of green development, increasing from 0.358 in 2010 to 0.379 in 2020. TIS contributes the most to the green development of the HCUA. IGD and RECC show a fluctuating trend of first rising and then falling, and UGC continues to develop with the improvement of municipal and transportation infrastructure.
2. In terms of the spatial distribution of the GDI level, the HCUA exhibits a patchy clustering differentiation feature that spreads from the central area to the surrounding areas with a “high in the south and low in the north” pattern. Changchun, Harbin, and Daqing form an “inverted triangle” structure in geographical location, driving the green development of adjacent areas. Technological and educational resources are concentrated in the central part of the urban agglomeration and gradually spread to the surrounding areas, and the spatial difference of RECC is not significant.
3. In the GDI and ranking of each city, Changchun and Harbin are much higher than other cities, at a relatively high level of green development. Jilin and Daqing are cities with an average level of green development. The GDI of the remaining cities is relatively low, with significant fluctuations in ranking. Liaoyuan and Songyuan have increased by four and two places, respectively, while Mudanjiang has dropped from fifth place to tenth place, and Siping has risen from last place to eighth place, showing certain potential for green development.
4. The importance and priority of the drivers differ among cities with different levels of green development. TIS is key to maintaining the leading position of cities with a high level of green development. At the same time, the continued promotion of TIS should be accompanied by a focus on the protection of the environment and the maintenance of environmental sustainability. While, cities with lower level are lagging behind in TIS and IGD, which need to make greater breakthroughs in RECC and UGD. All in all, upgrading the support of TIS will help high-level cities continue to lead, at the same time, it is also one of the ways for low-level cities to improve. The Harbin-Changchun urban agglomeration should strengthen environmental governance through rational allocation of resources in order to achieve a higher level of green development.
5. Focus on unbalance problem of the ecological and economic of the green development of urban agglomerations, the relevant data of HCUA show that both core cities and other cities still have a large space for ecological and economic development, and the developed speed of urban ecological environment and economic are out of sync, so it is necessary to promote the optimization and adjustment of economic and industrial structure according to different urban basic conditions. Through talent gathering, scientific and technological innovation and other aspects to promote the transformation of the industry to "two low and two high", in order to support the realization of urban green development goals.

### Recommendations

Based on the above conclusions, this study has the following recommendations:

1. Both the science and technology innovation environment and the traditional industrial pattern need to be upgraded in two aspects. On the one hand, according to the current development situation of each city, establish differentiated science and technology innovation strategies with the guidance of relevant policies. Specifically, continue to increase the construction of major science and technology infrastructure, which is conducive to linking scientific and technological achievements such as patents to green development. Technological innovation in relatively lagging cities relies on expanding the degree of regional openness. On the other hand, to achieve sustainable and efficient green development, the transformation of resource-based cities in HCUA is necessary with the premise of improving the resource utilization rate.
2. There are spatial, economic, and administrative barriers in the HCUA that lead to varying significantly and lack of synergistic cooperation of GDI among cities. The core cities should make full use of their location advantages to radiate and drive the synergistic development among cities in HCUA. Relying on railroad transportation to promote the flow of production factors, each city based on its characteristics to consolidate regional cooperation, cultivate sub-growth pole cities of Jilin, Daqing, and Suihua, to promote the formation of a new spatial pattern, and improve the benefits of green development.

## Data Availability

The data in this article were collected from the “China Urban Statistical Yearbook (2011–2021)”, “China Urban Construction Statistical Yearbook (2011–2021)”, City National Economic and Social Development Statistical Bulletin, and the data center website of the Chinese Academy of Sciences Resource and Environment Science (https://www.resdc.cn/). The data on indicators such as the number of patents authorized per 10,000 people and the number of major science and technology infrastructures were calculated based on the results obtained from the website of the National Intellectual Property Administration (https://www.cnipa.gov.cn/).
